# Aryl hydrocarbon receptor: a multifaceted environmental sensor in liver homeostasis and disease

**DOI:** 10.3389/fmed.2026.1801871

**Published:** 2026-05-08

**Authors:** Yanyan Jing, Xuying You, Yiming Feng, Suguo Wu, Kunning Wang, Hongxia Yuan, Junjian Liu

**Affiliations:** 1 College of Traditional Chinese Medicine, Tianjin University of Traditional Chinese Medicine, Tianjin, China; 2 Basic Research Center for Gastroenterology, Tianjin University of Traditional Chinese Medicine, Tianjin, China; 3 Tianjin NanKai Hospital, Tianjin Medical University, Tianjin, China; 4 Tianjin NanKai Hospital, Tianjin University of Traditional Chinese Medicine, Tianjin, China; 5 Faculty of Medicine, ITCWM Medical Center, Tianjin University, Tianjin, China

**Keywords:** aryl hydrocarbon receptor, dual role, hepatic fibrosis, liver injury, oxidative stress

## Abstract

The aryl hydrocarbon receptor (AhR) functions as a pivotal integrator of environmental and metabolic signals, playing a complex and highly context-dependent dual role in liver homeostasis and injury. On one hand, by sensing exogenous toxins or specific endogenous ligands, it can mediate mitochondrial dysfunction, oxidative stress, DNA damage, and aberrant metabolic activation, thereby driving hepatocyte apoptosis, steatosis, and inflammatory responses, which contributes to the initiation and progression of liver damage. On the other hand, AhR can also elicit hepatoprotective effects by responding to distinct ligand signals. These protective mechanisms include the inhibition of activation and fibrogenesis in hepatic stellate cells, the induction of anti-inflammatory phenotypes in immune cells, and the regulation of bile acid and lipid metabolic homeostasis. This review aims to summarize recent advances in understanding the role of AhR in liver injury, with a focus on dissecting the underlying mechanisms for its seemingly paradoxical functions. The insights provided herein are expected to offer a theoretical foundation and future research directions for exploring AhR-targeted intervention strategies in liver diseases.

## 1 Introduction

The aryl hydrocarbon receptor (AhR) has evolved from its classic toxicological role as the “dioxin receptor” into a complex homeostatic regulator and a pivotal hub in disease modulation. Its function is particularly prominent in the liver, a central metabolic organ, extending far beyond the simple regulation of xenobiotic metabolism. AhR constitutes a signaling integration platform, the activity of which is dynamically regulated by exogenous pollutants, dietary molecules, microbial metabolites, and endogenous ligands (Table 1) (1, 2). Consequently, it outputs biological effects that are highly dependent on the specific tissue microenvironment and the nature of the activating ligand. This profound context-dependency forms the fundamental basis for understanding the dual—protective

**TABLE 1:** Ligand- and cell type-specific protective and disruptive roles of the Aryl hydrocarbon receptor (AhR) in liver pathophysiology.

Effect	Ligand	Cell type	Mechanism
Disruptive	TCDD	HepG2	Promotes liver fibrosis development by inducing EMT via the AhR/Snail2 signaling pathway (3)
		Hepa1c2c7	Induces mitochondrial dysfunction by inhibiting the interaction between AhR and the ATP5α1 subunit, leading to mitochondrial membrane hyperpolarization (4)
	ITE	HSC	Attenuates liver fibrosis by inhibiting HSC activation
	BaP	HepG2	Induces intracellular lipid deposition in hepatocytes (5)
		Hepa 1c1c7	Induces oxidative stress (6)
	High-fat diet/FFA	HepG2	Promotes hepatic lipid synthesis and accumulation by enhancing miR-132 expression, leading to downregulation of SIRT1 and subsequent upregulation of lipogenic genes (7)
	Lenvatinib	HuH7 Hep3B	Confers lenvatinib resistance in HCC cells by promoting AREG expression, thereby activating the EGFR-ERK1/2-CyclinD1 signaling pathway (8)
	Cl-PAHs	H4IIE	Induces DNA damage in a dose-dependent manner (7)
	HCV	Huh-7	Increases lipid accumulation and HCV viral assembly via AhR-CYP1A1 (9)
	3-MC	Hepa 1c1c7	Induces oxidative stress (6)
	B[a]P-7,8-dione	Hepa1c1c7 Hepa1c1c4 Hepa1c1c12	Leads to DNA single-strand breaks by binding AhR, translocating to the nucleus, and generating ROS (10)
	β-NF	Hepa1c1c7	Induces oxidative stress (6)
	CH-223191	AML12	Induces mitochondrial dysfunction by reducing mitochondrial respiration rate, membrane potential, and utilization of tricarboxylic acid cycle substrates (11)
Protective	YH439	mHSCs	Alleviates mouse liver fibrosis by selectively promoting ferroptosis in mHSCs without inducing ferroptosis in hepatocytes (12)
	Kyn	AML12	Maintains liver mitochondrial homeostasis by regulating BNIP3 expression. AhR deficiency leads to mitochondrial dysfunction, increased ROS production, and suppressed mitophagy (11)
	3-MC	HepG2	Alleviates cholestasis, oxidative stress, and inflammation-induced liver injury by upregulating MRP4 expression (13)
	DIM	HSC-T6 HSC	Attenuates liver fibrosis by downregulating miR-21 expression, inhibiting the TGF-β signaling pathway, and suppressing HSC activation (14)
	OPZ	HepG2 PHH	Alleviates cholestasis, oxidative stress, and inflammation-induced liver injury by upregulating MRP4 expression (13)
	4-PBA	HepG2	Alleviates cholestasis, oxidative stress, and inflammation-induced liver injury by upregulating MRP4 expression (13)
	CH-223191	Huh-7	Reduces lipid accumulation and HCV viral assembly via the AhR-CYP1A1 axis (9)
		PMH	Alleviates HHcy-induced hepatic steatosis by inhibiting the AHR/CD36 pathway (15)
	Flutamide	Huh-7	Reduces lipid accumulation and HCV viral assembly via the AhR-CYP1A1 axis (9)

**FIGURE 1:**
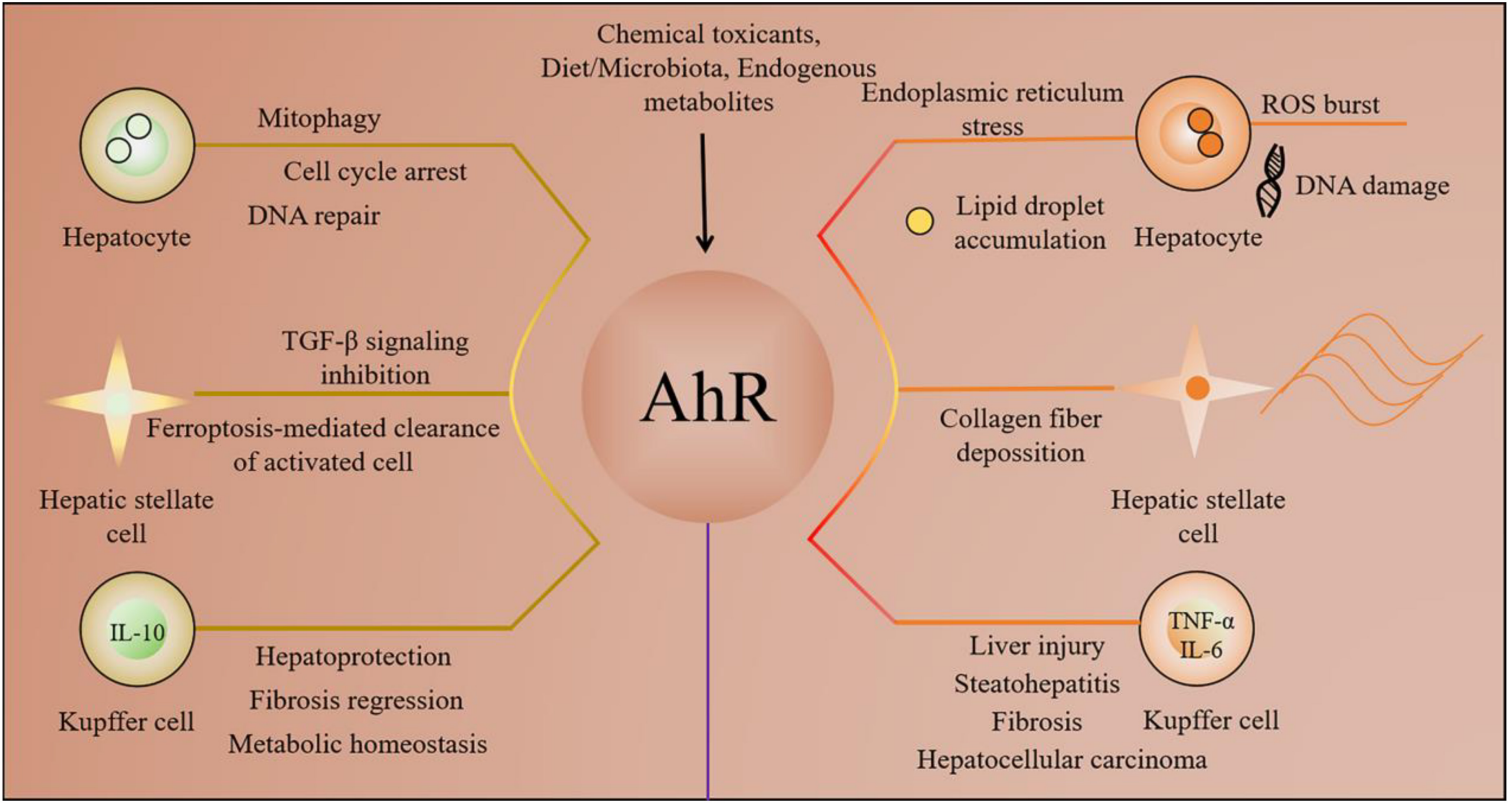
Schematic overview of the dual roles of the aryl hydrocarbon receptor in liver pathophysiology.

HSC, hepatic stellate cells; EMT, epithelial-mesenchymal transition; FFA, free fatty acids; PMH, primary mice hepatocytes; PRH, primary rat hepatocytes; 3-MC, 3-methylcholanthrene; PHH, primary human hepatocytes.

or detrimental—role of AhR in liver injury. The underlying mechanisms involve cell type-specific expression profiles of coregulators and the reprogramming of epigenetic modifications. This review aims to dissect the multifaceted roles of AhR in the liver, focusing on its function as a molecular hub coordinating stress adaptation, metabolic reprogramming, and immune regulation. Furthermore, it explores how the dysregulation of AhR drives liver injury and discusses how the precise targeting of the AhR signaling pathway may open novel avenues for the prevention and treatment of liver diseases. A schematic overview of the major protective versus disruptive pathways mediated by AhR in different hepatic cell types is provided in Figure 1.

## 2 Multidimensional signal perception and integration: context - dependency of AhR activity

The functional complexity of the aryl hydrocarbon receptor (AhR) stems from its broad and diverse ligand repertoire and its tissue-specific expression. In recent years, beyond classic dioxin-like compounds, emerging contaminants such as polyhalogenated carbazoles and polychlorinated diphenyl sulfides have also been identified as AhR activators (1, 16). In the liver, AhR is exposed not only to intestinally derived ligands entering via the portal vein (e.g., indole, indole-3-acetic acid, indole-3-propionic acid) but also responds directly to signaling molecules generated by hepatic metabolism itself (e.g., kynurenine, bilirubin metabolites) (17–19). This dual-exposure mode maintains hepatic AhR in a state of persistent “ligand exposure,” resulting in a significantly higher functional baseline compared to other organs. AhR signaling output is not a simple “on/off” switch but rather a graded and multidimensional response spectrum. This context-dependency is primarily manifested at the following levels:

### 2.1 Ligand properties determine signal quality

Subtle differences in the binding interactions between different ligands and AhR lead to nuanced conformational changes in the AhR-ARNT complex. This, in turn, enables the selective recruitment of distinct transcriptional coactivators (e.g., p300/CBP, SRC-1) or corepressors (e.g., SMRT, NCOR), ultimately regulating differential gene expression profiles. For instance, TCDD, acting as a high-affinity and poorly metabolized ligand, tends to induce a persistent and robust canonical AhR signal, driving massive *CYP1A1* expression and cell cycle arrest (3, 20, 21). In contrast, certain dietary indole derivatives (e.g., indole-3-carboxaldehyde, indole-3-acetic acid) exhibit weaker binding affinity and rapidly metabolized by CYP1A1, resulting in transient AhR activation. This transient nature, along with ligand-induced distinct conformational changes in the AhR-ARNT complex, skews the transcriptional program toward genes involved in immune regulation and tissue homeostasis (e.g., IL-22) (22–24).

### 2.2 Tissue and cell-type specificity

The downstream effects of AhR vary dramatically across different cell types. In hepatocytes, AhR primarily functions as a sensor of metabolism and oxidative stress; in hepatic stellate cells (HSCs), it acts as a key switch regulating activation and fibrogenesis (25); while in Kupffer cells, it is deeply involved in inflammatory polarization and immune regulation. These disparities originate from the extensive crosstalk between the AhR signaling pathway and other intrinsic signaling networks inherent to each cell type. For example, in hepatocytes, AhR extensively interacts with the nuclear factor erythroid 2-related factor 2 (Nrf2) antioxidant pathway; in HSCs, with the transforming growth factor-β (TGF-β) pathway; and in immune cells, with nuclear factor-κB (NF-κB) and signal transducer and activator of transcription (STAT) family members (26–28).

### 2.3 Dynamic regulation by pathophysiological status

In a healthy liver, AhR activation may predominantly participate in maintaining basal metabolic homeostasis and immune tolerance. However, under pathological conditions such as inflammation, fibrosis, or hepatocellular carcinoma (HCC), the hepatic microenvironment undergoes drastic changes (e.g., elevated inflammatory cytokines, hypoxia, extracellular matrix remodeling). Consequently, AhR’s expression level, subcellular localization, and interacting protein network can undergo adaptive alterations. For instance, during chronic inflammation, cytokines like IL-6 can upregulate AhR expression via the STAT3 signaling pathway. Simultaneously, the inflammatory milieu may alter the generation and distribution of AhR ligands, forming complex feedback loops that shift its function from homeostasis maintenance toward either promoting or suppressing disease progression (29).

In addition to ligand and cell-type specificity, AhR signaling is also modulated by sex and species differences. For instance, female mice exhibit higher basal AhR expression in the liver and are more susceptible to TCDD-induced hepatotoxicity than males, a difference attributed to estrogen receptor cross-talk and sex-specific regulation of CYP1A enzymes (30, 31). Moreover, significant species differences exist in AhR ligand affinity and downstream responses between rodents and humans, underscoring the need for caution when translating findings from animal models to human liver diseases (32, 33).

Therefore, the intensity, duration, and transcriptional consequence of AhR signaling are by no means static but are strictly constrained by context. The chemical nature of the ligand, the dose and duration of exposure, the type of target cell, and the pathophysiological state of the tissue collectively constitute a dynamic regulatory network that determines whether AhR activation leads to protective adaptation or pathogenic injury.

## 3 Core organelle function and cell fate determination

The aryl hydrocarbon receptor (AhR) profoundly influences the functions of core organelles that determine cell fate, converting environmental signals into cellular life-or-death decisions through direct and indirect mechanisms.

### 3.1 AhR-mediated mitochondrial quality control: from oxidative stress to mitophagy in hepatocytes

Aryl hydrocarbon receptor activation can significantly impact mitochondrial function and integrity. On one hand, it can induce the overproduction of mitochondrial reactive oxygen species (mtROS). This process involves AhR-mediated overexpression of CYP1A1, whose enzymatic reaction by-products directly generate ROS or indirectly cause ROS leakage by interfering with mitochondrial electron transport chain complex function (34). Excessive mtROS attacks mitochondrial lipids, proteins, and mtDNA, leading to the collapse of mitochondrial membrane potential, impaired ATP synthesis, and irreversible opening of the mitochondrial permeability transition pore (mPTP), thereby releasing pro-apoptotic factors like cytochrome c and initiating the intrinsic apoptotic pathway (34, 35). Studies indicate that AhR activation can regulate mitochondrial DNA stability and influence mitochondrial biogenesis in the liver. For instance, AhR has been shown to modulate the expression of protein phosphatase 2 regulatory subunit-bdelta (Ppp2r2d) and nuclear respiratory factors, thereby affecting mitochondrial mass and function in hepatocytes (36, 37). Furthermore, TCDD-induced mitochondrial oxidative stress and DNA damage are AhR-dependent and may be mediated through alterations in the mitochondrial thiol redox status in mouse liver (38). On the other hand, AhR participates in the initiation of mitophagy by regulating the expression of genes such as *BNIP3* in hepatic cells (4, 11). BNIP3, as a mitochondrial outer membrane receptor, anchors damaged mitochondria to autophagosomes. AhR regulation of *BNIP3* may represent a compensatory mechanism for cells to cope with AhR-induced mitochondrial damage, aiming to clear dysfunctional mitochondria and maintain overall cellular health. However, when damage exceeds the clearance capacity of autophagy, the cell proceeds toward death. Additionally, AhR may also directly interact with mitochondrial-associated proteins via transcription-independent mechanisms to rapidly modulate their function (39). This comprehensive intervention in mitochondrial biogenesis, function, and quality control positions AhR as a key regulator determining whether to repair or eliminate damaged mitochondria during metabolic stress or toxic injury.

### 3.2 Coordination of ER homeostasis and the unfolded protein response

Aryl hydrocarbon receptor signaling is intricately interconnected with the endoplasmic reticulum stress (ERS) response in the liver. Previous studies have reported that in hepatocytes, TCDD activates AhR, leading to abnormal protein processing, induction of ERS, and synergistic action with oxidative stress to cause hepatocyte injury (40). Despite these findings, direct evidence for a unified AhR-ERS regulatory axis in the liver remains fragmentary. Most existing studies have focused on non-hepatic tissues (e.g., lung, intestine), where AhR clearly induces ERS through ROS production, calcium dysregulation, and modulation of the unfolded protein response (41–43). However, whether these same mechanisms operate in hepatocytes, and how they integrate with the well-established roles of AhR in hepatic oxidative stress, DNA damage, and metabolic reprogramming, represents a major knowledge gap. Future research urgently needs to employ hepatocyte-specific AhR knockout mice, ERS reporter models, and liver organoid systems to determine whether AhR directly regulates hepatic ER homeostasis and to elucidate whether this axis drives protective or detrimental responses in a toxicant-specific context.

### 3.3 Shaping genome architecture and repair capacity

Aryl hydrocarbon receptor participates in the maintenance of genomic stability through its dual impact on the DNA damage response. Its classic pathogenic role involves mediating genotoxicity: AhR-mediated expression of enzymes like CYP1A1 and CYP1B1 metabolizes polycyclic aromatic hydrocarbons such as benzo[a]pyrene, generating highly reactive electrophilic epoxides and radical cations. These metabolites can directly form covalent adducts with DNA bases or indirectly cause DNA single/double-strand breaks and base oxidation via massive ROS production (10, 44–46). However, AhR activation can also trigger protective responses. Research has found that AhR activation can induce G1/S phase cell cycle arrest, providing a potential time window for DNA repair (10, 45). This arrest involves mechanisms such as AhR forming a complex with the RB1 protein to inhibit E2F transcription, downregulating cyclins (Cyclin D1, Cyclin A), and upregulating the cyclin-dependent kinase inhibitor p27 (47, 48). Interestingly, this arrest exhibits a self-limiting mechanism: induced enzymes like CYP1A1 can accelerate the degradation of their ligands (e.g., metabolic clearance of TCDD), thereby providing feedback to attenuate persistent AhR activation and allowing cell cycle progression to resume (49–51). This intricate feedback regulation aims to balance the needs of DNA damage repair and cell proliferation. Furthermore, AhR also exerts regulatory effects on the DNA repair pathways themselves. Studies indicate that AhR activation impairs nucleotide excision repair and base excision repair functions, potentially by downregulating repair enzyme expression or competitively occupying necessary transcriptional cofactors (52, 53). Therefore, AhR plays a context-dependent dual role in the DNA damage and repair network: it is both a generator of damage (via metabolic activation) and a modulator of the damage response (by influencing the cell cycle and repair capacity). The dynamic balance between these opposing actions ultimately determines whether AhR acts as to suppress or promote genomic instability, with profound implications for the development and progression of HCC. In HCC, AhR expression is often dysregulated, and its activation can either inhibit tumor growth by inducing cell cycle arrest or promote malignancy by enhancing survival pathways and metabolic reprogramming, depending on the ligand and tumor micro-environment (10).

## 4 Global reprogramming of metabolic homeostasis

Aryl hydrocarbon receptor serves as one of the central regulators of the hepatic metabolic network, and its dysfunction is a key driver of metabolic liver diseases.

### 4.1 Ligand-directed regulation of lipid metabolism

Aryl hydrocarbon receptor’s regulation of hepatic lipid metabolism exhibits characteristic ligand-dependent heterogeneity, a vivid manifestation of its context-dependency. In the context of non-alcoholic fatty liver disease (NAFLD), AhR activation can promote the expression of the fatty acid transporter CD36, increasing hepatic uptake of circulating free fatty acids and thereby exacerbating lipid input load (15). However, reports on its regulatory direction over hepatic *de novo* lipogenesis (DNL) are inconsistent, likely stemming from differences in ligands, models, and disease stages used across studies. For example, AhR agonists like β-naphthoflavone or the endogenous ligand indole-3-acetic acid have been reported to downregulate the expression of key synthetic genes such as acetyl-CoA carboxylase 1 (ACC1), fatty acid synthase (FASN) and stearoyl-CoA desaturase (SCD1), thereby ameliorating hepatic lipid deposition (54). The underlying mechanisms may involve AhR-mediated inhibition of sterol regulatory element-binding protein 1c (SREBP-1c) transcriptional activity or interference with upstream regulatory pathways of lipid synthesis, such as the liver X receptor (LXR). Conversely, other studies suggest that under certain conditions (e.g., high-fat diet combined with specific ligand exposure), AhR signaling may also drive lipogenesis by upregulating SREBP-1c or directly activating the promoters of lipogenic enzyme genes (54, 55). Furthermore, AhR can influence the fatty acid β-oxidation process. This “ligand-directed” regulation allows AhR to function as a metabolic switch, the direction of which depends on the specific chemical instruction it receives. This provides an intriguing theoretical basis for intervening in NAFLD through dietary supplementation (e.g., indole-3-carbinol from cruciferous vegetables) or modulation of the gut microbiota to generate specific AhR ligands.

### 4.2 Modulation of bile acid metabolism and the gut-liver axis

Aryl hydrocarbon receptor constitutes a pivotal node in the chemical communication between the liver and the intestine, known as the “gut-liver axis.” In pathological processes such as cholestasis, AhR activation can induce the expression of various Phase I and II metabolizing enzymes. Notably, although CYP3A4 is primarily regulated by CAR and PXR, emerging evidence indicates that AhR can also transactivate the CYP3A4 promoter through xenobiotic response elements (XREs) or via cross-talk with other nuclear receptors (56). Additionally, AhR promotes the expression of UDP-glucuronosyltransferases (UGTs), such as UGT1A1, facilitating the hydroxylation and conjugation of hydrophobic bile acids, thereby enhancing their water solubility and excretion (57, 58). Concurrently, AhR can regulate the expression of bile acid transporters. For instance, it upregulates multidrug resistance-associated protein 4 (ABCC4/MRP4), which pumps bile acids and glutathione conjugates out of cells, potentially altering the bile acid pool composition and reducing the concentration of potent FXR agonists, subsequently inhibiting the FXR signaling pathway (59, 60). Studies have shown that in a 3,5-diethoxycarbonyl-1,4-dihydrocollidine (DDC) diet-induced cholestasis model, AhR upregulation exerted a clear protective effect, manifested by decreased serum bilirubin and alkaline phosphatase levels, along with reduced intrahepatic bilirubin pigment deposition (61). The protective mechanisms of AhR may include: (1) promoting the metabolic clearance of toxic bile acids; (2) reducing total bile acid synthesis by negatively regulating the rate-limiting enzyme CYP7A1 in bile acid synthesis; and (3) finely tuning bile acid homeostasis through synergistic or antagonistic interactions with nuclear receptors like FXR. By continuously sensing gut microbiota-derived metabolites (e.g., secondary bile acids, indole derivatives) and adjusting hepatic strategies for bile acid synthesis, modification, and excretion accordingly, AhR perfectly fulfills its role as a chemical translator of the gut-liver axis.

### 4.3 Dynamic remodeling of xenobiotic metabolic capacity

As a core transcriptional regulator of Phase I metabolizing enzymes such as CYP1A1, CYP1B1, and CYP1A2, the activation state of AhR directly determines the hepatic “metabolic fingerprint,” profoundly influencing the rate and pathway of biotransformation for exogenous drugs, environmental toxins, and certain endogenous compounds (62, 63). This regulation does not occur in isolation but is integrated with the organism’s overall status. For example, inflammatory cytokines (e.g., IL-6, TNF-α) can suppress CYP450 expression by downregulating hepatocyte nuclear factor 4α (HNF4α) or inducing miRNAs. AhR signaling may interact with these inflammatory pathways, leading to complex alterations in drug metabolism during infections or autoimmune liver diseases (6, 64). Furthermore, hypoxia and hormonal changes can also modulate AhR activity. In pathological states such as HCC, AhR signaling often undergoes reprogramming, which may lead to a global downregulation of drug-metabolizing enzymes or abnormal overexpression of specific subtypes. For example, CYP1A1 is frequently upregulated in HCC tissues in an AhR-dependent manner, contributing to the metabolic activation of pro-carcinogens and resistance to chemotherapeutic agents (65). Conversely, downregulation of CYP3A4 via AhR cross-talk with inflammatory pathways can alter the pharmacokinetics of many anticancer drugs (66). These changes not only affect the efficacy and toxicity of chemotherapeutic drugs but may also alter the profile of endogenous metabolites in the tumor microenvironment, thereby influencing tumor progression. Understanding thes AhR-mediated alterations in drug metabolism holds critical implications for personalized medicine, particularly in predicting drug-induced liver injury and optimizing therapeutic regimens for HCC patients.

## 5 Shaping the immune microenvironment and tissue remodeling

The AhR plays a complex and central role in maintaining hepatic immune tolerance and in the inflammation-fibrosis cascade, with its function exhibiting high heterogeneity across different immune and stromal cells.

### 5.1 Fine-tuning of immune cell function

Aryl hydrocarbon receptor acts as a “multifunctional modulator” for hepatic immune cells, characterized by its dual nature. In Kupffer cells, the resident macrophages of the liver, AhR activation can promote pro-inflammatory cytokines production (e.g., TNF-α, IL-1β, IL-6) in response to lipopolysaccharide or toxicants, contributing to acute liver injury. Conversely, AhR signaling in Kupffer cells can also drive IL-10 expression via non-genomic Src-STAT3 pathways, limiting excessive inflammation (6, 16, 29). In hepatic dendritic cells, AhR modulates T cell polarization, favoring either Th17 or regulatory T cell (Treg) differentiation depending on the ligand. In liver-resident lymphocytes such as γδ T cells and innate lymphoid cells, AhR is essential for IL-22 production, which promotes tissue repair but can also exacerbate fibrosis in chronic settings (67) . These context-dependent functions position AhR as a precise “tuner” of hepatic immune responses. For instance, in a silica-induced pulmonary inflammation model, AhR global knockout mice exhibited more severe early acute inflammation but attenuated later-stage fibrosis, revealing that AhR may exert opposing effects at different stages of inflammation (68). Although direct evidence in the liver is still emerging, similar temporal specificity has been observed in models of alcoholic liver disease and non-alcoholic steatohepatitis (NASH), where AhR promotes early inflammatory responses but later contributes to fibrosis resolution or progression depending on the ligand milieu.

### 5.2 The bidirectional regulatory hub in fibrogenesis

Aryl hydrocarbon receptor’s function in HSCs exemplifies its context-dependency in tissue remodeling and represents a key therapeutic target for anti-fibrotic strategies. During the transdifferentiation of HSCs from a quiescent state to an activated myofibroblast-like phenotype, the expression and function of AhR undergo dynamic changes. Toxic exogenous ligands, such as TCDD, can directly upregulate the transcription of fibrotic markers like collagen type I alpha 1 chain (COL1A1) and α-smooth muscle actin (α-SMA) through AhR-dependent mechanisms. They also promote the release of inflammatory factors, thereby driving HSC activation and excessive extracellular matrix (ECM) deposition, ultimately inducing liver fibrosis (3). The insensitivity of AhR-knockout mice to the pro-fibrotic effects of TCDD confirms its necessity. However, the other side of the story is that certain endogenous ligands [e.g., 2-(1’H-indole-3’-carbonyl)-thiazole-4-carboxylic acid methyl ester, ITE] or well-designed synthetic ligands (e.g., YH439) can exert potent anti-fibrotic effects by activating AhR. The underlying mechanisms are highly innovative and selective. For example, YH439 activation of AhR upregulates multidrug resistance protein 1 (MRP1/ABCC1), promoting the efflux of reduced glutathione (GSH) from activated HSCs. This leads to the depletion of intracellular antioxidant capacity, accumulation of lipid peroxides, and ultimately triggers ferroptosis specifically in activated HSCs, while sparing normal hepatocytes (12). This provides a novel strategy for the precise elimination of pathogenic cells. Additionally, studies have confirmed that in quiescent HSCs, AhR actually plays an inhibitory role. Its high expression and activation enable it to interact with the Smad3 protein, inhibiting TGF-β1-induced transcription of pro-fibrotic genes (25). HSC-specific AhR knockout mice developed more severe fibrosis in carbon tetrachloride (CCl4) or bile duct ligation models, whereas hepatocyte- or Kupffer cell-specific AhR knockout did not yield this phenotype. This strongly demonstrates that the intrinsic anti-fibrotic function of AhR in HSCs is cell-autonomous and specific (25). This phenomenon, where opposing functions arise within the same cell type depending on the ligand, underscores the plasticity of the AhR signaling pathway and its immense potential as a drug target. The future challenge and opportunity lie in developing “selective AhR modulators” that can precisely target AhR in HSCs and mimic its protective conformational changes.

## 6 AhR in HCC: a double-edged sword in tumorigenesis

The role of AhR in HCC is complex and context-dependent. On one hand, AhR activation by environmental carcinogens such as benzo[a]pyrene promotes DNA adduct formation and genomic instability, contributing to tumor initiation (69, 70). On the other hand, AhR can exert tumor-suppressive effects by inducing G1/S cell cycle arrest and promoting apoptosis in certain HCC cell lines (71). In established tumors, AhR signaling often becomes dysregulated; elevated AhR expression correlates with poor prognosis and chemoresistance in some HCC cohorts, while in others it is associated with better outcomes. These discrepancies likely reflect differences in ligand availability, tumor microenvironment, and cross-talk with oncogenic pathways such as Wnt/β-catenin and EGFR (8). Emerging evidence also implicates AhR in modulating the tumor immune microenvironment by affecting the recruitment and function of myeloid-derived suppressor cells and regulatory T cells (72). A deeper understanding of AhR’s cell-type- and stage-specific functions in HCC will be critical for developing AhR-targeted therapies that avoid unintended pro-tumorigenic effects.

## 7 Future perspectives: toward precision AhR-targeted therapy for liver diseases

The multifaceted role of the AhR in liver injury presents both a challenge and an opportunity. Its core advantage as a therapeutic target lies in its widespread tissue expression and potent signal integration capacity; the primary challenge resides in achieving precise modulation to avoid its detrimental effects while amplifying its protective functions. Future research should focus on the following frontiers, with particular attention to specific liver disease entities including NAFLD/NASH, liver fibrosis, cholestatic diseases, and HCC.

### 7.1 Deciphering cell-type-specific AhR networks in distinct liver diseases

The functional outcome of AhR activation varies dramatically across different hepatic cell types and disease contexts. Future studies should utilizing cell-type-specific AhR-knockout or reporter mouse models (e.g., targeting hepatocytes, HSCs, Kupffer cells, liver sinusoidal endothelial cells) combined with single-cell sequencing, chromatin immunoprecipitation sequencing (ChIP-seq), and proteomics to comprehensively map AhR’s cell-specific binding sites, transcriptional regulatory networks, and protein interaction profiles under different physiological and pathological states.

In NAFLD/NASH, such approaches could reveal how AhR in hepatocytes versus macrophages differentially regulates lipid accumulation, insulin resistance, and inflammatory cascades. In liver fibrosis, dissecting AhR’s interactome in quiescent versus activated hepatic stellate cells may uncover mechanisms that govern the transition from pro-fibrotic to anti-fibrotic signaling. In HCC, mapping AhR networks in tumor cells versus tumor-infiltrating immune cells (e.g., myeloid-derived suppressor cells, tumor-associated macrophages, cytotoxic T cells) could identify critical nodes that influence immune evasion and therapeutic resistance. Particular attention should be paid to how AhR recruits distinct epigenetic modifiers (e.g., histone acetyltransferases, deacetylases, methyltransferases) to regulate gene expression in a cell- and disease-specific manner, which may represent the underlying mechanism for its functional diversity.

### 7.2 Rational design of functional ligands for disease-stage-specific intervention

Structural biology studies of AhR-ligand complexes are needed to gain deeper insights into how conformational differences induced by distinct ligands lead to biased downstream signaling. Building on this knowledge, computer-aided drug design and synthetic chemistry can be employed to develop novel AhR modulators with tailored properties.

For NAFLD/NASH, ligands that selectively promote fatty acid oxidation while suppressing *de novo* lipogenesis in hepatocytes, or that skew Kupffer cells toward an anti-inflammatory M2 phenotype, would be highly desirable. For liver fibrosis, the identification of ligands that selectively induce ferroptosis in activated hepatic stellate cells—such as YH439—represents a promising strategy, and further optimization of such compounds could yield potent anti-fibrotic agents. For cholestatic diseases, AhR ligands that enhance bile acid detoxification without disrupting enterohepatic circulation warrant investigation. For HCC, the development of ligands that induce tumor cell cycle arrest or sensitize tumors to immune checkpoint inhibitors, while avoiding the promotion of cancer stem cell populations, represents a critical therapeutic opportunity. Natural product libraries and gut microbiota metabolites serve as valuable resources for discovering novel lead ligands with favorable safety profiles.

### 7.3 Elucidating the dynamic role of AhR across human liver disease progression

Utilizing clinical samples (including liver tissues from diseases of various etiologies and stages) and organoid models (e.g., hepatocyte-organoids, cholangiocyte-organoids, or co-culture systems incorporating immune cells and stellate cells), longitudinal studies should investigate the dynamic alterations of AhR signaling throughout the entire spectrum of human disease progression.

For NAFLD, it is crucial to determine whether AhR activation contributes to the transition from simple steatosis (NAFL) to NASH, and whether this transition involves a shift from protective to pathogenic AhR signaling. In liver fibrosis, studies should clarify at which stage AhR activation in HSC transitions from an intrinsic anti-fibrotic brake to a pro-fibrotic driver under chronic injury conditions. In HCC, it is essential to delineate whether AhR acts as a tumor suppressor in early stages but becomes a pro-oncogenic factor in advanced disease, and how the tumor microenvironment modulates AhR function. This knowledge is essential for identifying the optimal therapeutic window AhR-targeted interventions and for developing stage-specific treatment strategies.

### 7.4 Developing cell-specific delivery strategies and combination therapies

Given the complex cellular composition of the liver and the potential for off-target effects, developing nanoparticle-based delivery systems capable of specifically targeting AhR ligands to desired cell types represents a key technological direction for enhancing efficacy and reducing systemic side effects. Examples include nanoparticles decorated with HSC-targeting peptides (e.g., cyclic peptides recognizing platelet-derived growth factor receptors) for anti-fibrotic therapy, or ligands targeting macrophage surface receptors (e.g., mannose receptors) for immunomodulation.

Furthermore, the potential of combining AhR modulators with existing therapeutic agents should be evaluated. In NASH, combining AhR ligands that promote metabolic benefits with anti-inflammatory agents (e.g., FXR agonists, GLP-1 analogs) could yield synergistic effects. In liver fibrosis, the combination of AhR-induced ferroptosis in activated HSCs with existing anti-fibrotic drugs may enhance fibrosis regression. In HCC, evaluating the combination of AhR modulators with immune checkpoint inhibitors (e.g., anti-PD-1/PD-L1) or tyrosine kinase inhibitors (e.g., sorafenib) could uncover novel strategies to overcome therapeutic resistance.

In conclusion, AhR has ascended from a marginalized toxicological receptor to a central regulatory node in liver physiology and pathology. By deciphering the complex signaling networks governed by ligand specificity, cellular context, and disease stage, and by leveraging cutting-edge technologies to develop precise intervention tools, we hold the promise of transforming AhR from a paradoxical biological puzzle into a programmable therapeutic switch. Targeting the AhR pathway not only provides a novel perspective for understanding the mechanisms of liver diseases-ranging from NAFLD/NASH and cholestatic diseases to liver fibrosis and HCC- but also may usher in a promising new era for the prevention and treatment of these conditions. The path forward lies in integrating cell-type-specific biology, rational ligand design, disease-stage-aware strategies, and innovative delivery technologies to unlock the full therapeutic potential of AhR modulation.
